# Responsiveness of a patient in a persistent vegetative state after a coma to weekly injections of autologous activated immune cells: a case report

**DOI:** 10.1186/1752-1947-6-6

**Published:** 2012-01-10

**Authors:** Barbara Fellerhoff, Barbara Laumbacher, Rudolf Wank

**Affiliations:** 1 Institute of Immunology, University of Munich, Goethestrasse 31, 80336 München, Germany; 2 Immunotherapy Research Center, Pettenkoferstrasse 8, 80336 München, Germany

## Abstract

**Introduction:**

An 82-year-old Caucasian woman had remained in a persistent vegetative state after a coma of seven months duration, which occurred after a stroke with hemiplegia, nine years previously. The persistent vegetative state could be reversed in part by weekly injections with activated immune cells. After therapy, our patient responded to commands in addition to regaining spontaneous movements of both arms and the ability to swallow. This is the first report on the treatment with activated immune cells of a patient in a persistent vegetative state after a coma.

**Case presentation:**

An 82-year-old Caucasian woman presented with a persistent vegetative state subsequent to a coma. She retained respiratory and autonomic functions. As contact was not possible, physiotherapy was passive. Her skin was yellowish, and our patient did not move by herself. Vomiting repeatedly resulted from tube feeding. After a once-weekly treatment with activated immune cells sampled from our patient's blood and activated *in vitro*, several of her functions gradually returned. Our patient opened her eyes in the requested direction and turned her head toward people entering the room. She 'supported' nursing efforts, as the nurse noted a loss of spastic motions. The strength in both her arms returned, and she spontaneously moved her arm on the side experiencing hemiplegia. After three months, our patient could stick out her tongue upon demand. Finally, the swallow reflexes of our patient started to return. However, tube feeding was continued, and our patient died after aspiration of vomit following a feeding.

**Conclusion:**

The success of treatment with autologous activated immune cells in this patient may have resulted from the production of neuroactive substances, such as neurotrophin-3 and brain-derived neurotrophic factor, by activated immune cells. The deterioration of our patient could be reversed, as demonstrated by the restoration of motor strength in her hemiplegic side. In addition, our patient was able to induce motor responses upon request. It seems reasonable to conclude that activated immune cells may improve the chronic vegetative state in some patients.

## Introduction

At the Institute of Immunology of the University of Munich, we have successfully treated several patients with psychiatric diseases with autologous activated immune cells (ACT). The treatment concept was based on previous observations of patients with persistent infections with *Chlamydophila *and the observed ability of immune cells to produce neuroactive substances [1-3]. The son of the patient in our case report had witnessed the tantrum of an autistic child and the subsequent dramatic improvements of the child after receiving activated immune cells [2]. He asked us whether we could help his mother. We agreed that we could and he discussed this with other family members. They encouraged him to proceed with treating his mother with activated immune cells.

### Case presentation

An 82-year-old Caucasian woman presented to our clinic with a persistent vegetative state after a coma. She was admitted at the age of 73 years, one year after a stroke, to the neurologic rehabilitation department of a Bavarian hospital. The diagnoses at that time included motor hemiparesis, motor aphasia, an inability to speak, an inability to swallow and an inability to actively move or react to contact attempts. She groaned continuously and on neurological examination, both her pupils were round and reacted directly and indirectly to light. It was not possible to examine her coordination because of an inability to stand or walk. In addition, she had no reaction on sensibility tests. A sensory aphasia was assumed.

Electroencephalography indicated a left-sided frontotemporal theta-delta state and no epilepsy-specific signs. Computed tomography indicated an extensive infarction and/or occlusion of the left middle cerebral artery and a widened left ventricle as well as a lowered density of her left basis pontis and brain stem. The reported laboratory values for her blood, liver and kidneys were in the normal range.

Therapeutic modalities instigated at that time by that hospital included a continuation of anticoagulation therapy which had started one year prior. A urinary infection that was discovered was treated with norfloxacin (based on resistance tests). Any logopedic or physiotherapeutic action was considered to be fortuitous, as only passive physiotherapy was possible. A feeding tube was inserted because of the patient's inability to swallow. She was dismissed from that hospital after six weeks for care at home. It was assumed that she would survive only a few months and that she should live the last months of her life in the home environment. The nursing was to be administered by her relatives, all farmers, with a nurse coming every day. Unless a special event necessitated an earlier visit, a family doctor visited every two weeks.

Nine years after her initial stroke, the 82-year-old woman had passed into a deep sleeplike state that had already lasted seven months, as the woman became our patient. Patient contact was not possible, and there were few vocal signs, such as groaning or cries. Her limbs had to be passively moved, as no active physiotherapy could be induced. She did not change position in the bed by herself, and active movements with her arms or legs were not observed. The only remaining signs of patient activity were vocal signs such as uttering of the same word, 'Maria', or occasional groaning. She was continuously administered anticoagulant therapy and artificial feeding. A search for microbial infections indicated serologically significant immunoglobulin G and immunoglobulin A titers against *Chlamydia trachomatis, Chlamydophila pneumoniae *and a suspected reactivation of Epstein-Barr virus

After isolation of immune cells from her peripheral blood, as described elsewhere [4], the immune cells were incubated *in vitro *with the OKT3 anti-CD3 antibody (ORTHOCLONE, muromonab-CD3 human T cell blocker, Janssen-Cilag, Neuss, Germany), which activates T lymphocytes. The stimulated T lymphocytes activate other immune cells, mainly monocytes and dendritic cells, and produce neuroactive substances [5]. These *in vitro*-activated mononuclear immune cells were administered by intramuscular injection. Alternatively, the activated immune cells were used to activate other not yet activated immune cells *in vitro*. These 'cascade primed' immune cells better recognize and eliminate infected cells and cancer cells [6]. As described previously for the treatment of patients with schizophrenia or depression [2], approximately 40 × 10^6 ^to 60 × 10^6 ^autologous activated immune cells were injected intramuscularly once per week.

**Figure 1:**
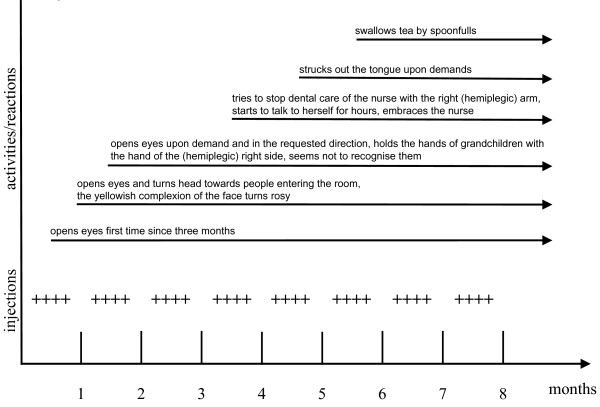
**shows the number of injections over a time period of eight months and resulting improvements in the patient's status**. Each "+" represents an injection. The activated autologous immune cells were administered weekly. Black arrows illustrate the respective point in time when a certain observation was made. All improvements persisted until the patient's death.

Several changes in the behavior of our patient could be observed after the initiation of ACT. After the first injections, our patient opened her eyes upon demand and moved them in the requested directions. Furthermore, our patient's eyes followed the person entering the room, and she was able to turn her head (Figure 1, month one). Other motor function improvements occurred more slowly, but our patient was able to change her side position in bed by herself and, after six weeks, she could grab for the bar of the bed with the hemiplegic-side hand (Figure 1, month two). Six months after beginning the immune cell therapy, a logopedist started training for the swallow reflex and soon claimed that our patient could swallow independently. The nurse had observed that our patient independently move her tongue during tooth brushing. Although our patient grabbed the hands of her grandchildren with both hands, held them for a lengthy period and looked at the children, this was not definite proof that she recognized them. Unfortunately, our patient died after a night-time tube feeding after aspirating vomit.

## Discussion

Our patient was nursed at home for nine years. She had to be turned on her side in the last seven months. In addition, no active movement was registered, and no patient contact was possible. She was tube fed. The first signs of new neural activities were only observed after the first weekly ACT injections (Figure 1, month one). Eye-opening was not considered a useful sign of improvement, but the autonomously turning of her head and eyes toward people entering the room was interpreted as a sign of initial improvement in sensory perception. This interpretation was supported by the repeated response to requests to move her eyes in a specific direction (Figure 1, month two). The motor strength of her arm on the unaffected side increased after a few weeks, and spontaneous movement of her arm on the hemiplegic side was observed after four months (Figure 1, month four). At that time, our patient could stick out her tongue upon request, a sign of both sensory and motor improvement. The logopedist initiated swallow reflex training and claimed after several sessions that the reflex was working (Figure 1, month six). However, the relatives and the nurse were not convinced of this progress, although the swallowing of a spoonful of tea seemed possible. In additon, the nurse noted one time in the care diary that she saw the patient swallowing after hearing the husband swallowing. During a session of night-time tube feeding, the patient vomited and then aspirated the vomit, which resulted in her immediate death.

There are rare examples of recovery in all stages of coma. In this patient, there was no clear evidence of recovery from the dementia state; however, signs of gradual improvement in her sensory perception and motor functions were observed. The gradual improvement accompanied the continuation of weekly injections with autologous activated immune cells. Whether this recovery would have continued with additional cell therapy cannot be answered. Activated immune cells can pass the blood brain barrier. If the assumption that the improvements in this patient were based on the ACT is accepted, then it is important to consider which mechanism might be responsible for the observed improvement in the function of nerve cells. It is possible that the supply of neuroactive substances by immune cells such as neurotrophin-3, brain-derived neurotrophic factor (BDNF) and others may 'restart' or improve synaptic activities, as they are known to enhance the release of neurotransmitters from neurons [7,8]. The participation of BDNF in the repair of nerve injuries in the periphery may also be observed in the central nervous system [9].

## Conclusion

Therapy with activated immune cells may improve the persistent vegetative state in some patients. The true value of ACT can be only ascertained in further studies.

## Consent

Written informed consent was obtained from the patient's son for publication of this case report and any accompanying images. A copy of the written consent is available for review by the Editor-in-Chief of this journal

## Competing interests

The authors declare that they have no competing interests.

## Authors' contributions

BF, BL and RW were involved in acquiring data and writing the manuscript; RW designed the concept of the treatment. All authors read and approved the final manuscript.
